# Indents

**Published:** 1909-04-15

**Authors:** 


### INDENTS.

**☞ Brief Articles of Technical or Scientific Value will receive notice and comment. Contributions invited.**

*Evans Museum and Institute.* The trustees of the proposed Evans Dental Museum, at Fortieth and Spruce streets, are almost ready to begin carrying out the plans of the millionaire dentist. In speaking of the outlook one of the trustees said: "The trustees have had conveyed to them property of a value that might be conservatively estimated at $1,000,000. The litigation is still pending as regards a small portion of this. It is believed, however, that it will be shortly settled favorably to the Museum. This property consists of real estate which will have to be converted into money before it becomes available for the purposes of the Institute. The trustees have, however, referred to the Executive Committee the formulation of plans for carrying out the purposes of the will. No definite progress will be made until all litigation is ended and the entire estate that is to come to the Museum finally assured. The committee, however, will preliminarily consider the question of an adequate structure to be used for the Museum or Institute." The relics of the famous American dentist are now in this city. They came packed in 137 cases and are now stored in safe deposit vaults awaiting the time when they may be transferred to their final places in the Museum. The jewels alone, many of them gifts from the dentist's royal patients, are valued at $50,000. —*Philadelphia Record.*

*Porcelain Crown with Cast Base.* An esthetic restoration of bicuspids and molars. The technique consisted of grinding roots one and one-half millimeters below the gum line on the buccal side and one millimeter on the palatal or lingual side. Enlarge the root canals for iridio-platinum pins, of a gage to suit the case. Cut the pins flush with the surface of the roots, and countersink the openings of the root canals one and one-half millimeters with inlay burs. Grind a detachable porcelain crown suitable for the case to approximately fit the root. Press wax on the end of the crown and against the root. Note where the openings are located. Heat the pins slightly and with pliers anchor to place. Press the pins, wax and tooth on to the root. Trim off all excess of wax, put in the sprue wire, and treat the case like an inlay, casting directly into the porcelain.

Care must be taken not to check the porcelain. If the operator desires, he can remove the porcelain crown before casting, thereby casting the base only. The crown is then cemented to the base.

It is the opinion of the clinician that such a crown properly adjusted will not split the roots, nor will the root decay at the junction of the root and base, and if broken it can be easily repaired by replacing the broken crown with another of the same mold. A record should be kept of the molds and the kinds of teeth used in such cases.—G. S. Schlegel, in March *Cosmos*.

*School-boys Neglect the Tooth Brush.* A class of forty boys averaging 10 years of age, was having a recitation on the the physiology of digestion. The use of the teeth, mouth, saliva, and gastric juices were considered, and the answers given showed the results of good teaching, but when the exercise was over there was left the feeling of incompleteness. What had the boys gained that was useful? Some knowledge of the processes of digestion. But how were they to make use of this knowledge? So far it was a general knowledge only. Could any part of it be made personal? Why, yes, the part the teeth play in the process of digestion might be made personal. Questions were then asked concerning the value of the teeth. What would happen if there were few teeth or none at all? What caused the loss of teeth? What were the evil results of diseased teeth? The boys were eager to answer these questions, edged out of seats, waved hands frantically, scowled at wrong answers, and when called upon to answer stood erect by the desk and proudly gave what they considered correct answers. To the final question, "How many of you boys have cleaned your teeth this morning?" there was but one response. One boy in a class of forty had cleaned his teeth that morning. The other boys sat dejectedly in their seats, ashamed. Further questioning showed that more than one-half of these boys made no use of the toothbrush at all, the others only occasionally. —*Iowa Health Bulletin.*

*A Useful Modification of the Anchorage Used in Expanding the Dental Arch.*

The object of this anchorage is to provide for the expansion of the entire arch, roots and crowns, as well as the alveolus in which they are implanted, and possibly for a widening of the nasal passage, by stimulating further development of the adjacent bones of the face. The marked changes in the facial expression which can be accomplished would seem to indicate that such a further development does take place. The construction is simple. Fit strong bands upon one or several molar teeth and upon the canine. To these bands, a connecting wire is soldered which curves upward between the gum and cheek to a point at or above the middle of the root like an inverted U, with the ends attached to the bands. To the summit of these strong rigid connecting arches or tubes are attached for the ends of the expansion arch, which by a bayonet-bend downward on each side may be made to pass around the arch low enough to permit ligaturing of the anterior teeth, and avoid irritating the frenum labium. With this device the ends of the roots may be expanded even more than the crowns, if it is desired, by carrying the loop and attached tube a little above the middle of the root, which can usually be done without irritation to the buccal tissues, as in the extremely contracted arches which demand this form of expansion the lips and cheeks are loose and flabby, with plenty of room for appliances. The square-tube and square-end expansion arch may be used in conjunction with this form of anchorage, if desired.—H. E. Kelsey, in March *Cosmos*.

*Chloroform vs. the Essential Oils as Gutta-percha Solvents.* Chloroform readily dissolves gutta-percha, some of the essential oils are also solvents, notably, eucalyptus oil and oil of cajuput. Chloroform quickly evaporates, leaving the gutta-percha in much the same condition as it found it; the oils, however, do not quickly evaporate. Chloropercha, a technical name for a solution of gutta-percha in chloroform, has long been used as a pulp canal filling, notwithstanding its many objectionable features. It must be kept in solution by constant additions of the solvent, it is "messy," it is difficult to pack well into the canals on account of its quickly thickening owing to the solvent evaporating, and requires much careful manipulation to ensure filling the canal solidly. It is repelled by moisture, and usually contracts, or fails to thoroughly fill the space owing to its becoming unworkable before this is accomplished. When oil of eucalyptus or cajuput is used the stock bottle is not needed. The oil is placed in the canals with a minim syringe and warmed by passing into it a heated instrument. This usually vaporizes or displaces a portion, the canals are again filled and a gutta-percha cone or point passed into the canals. In a few moments this softens, and may be packed solidly with a suitable instrument, and more gutta-percha added if necessary. It remains plastic until thoroughly packed and, if oil of cajuput is used, is not repelled should a little moisture be present, and it does not contract. A minute pellet of gold is frequently useful in forcing the gutta-percha well to the apex, it stays there as part of the filling. A little iodoform may be added to the oil in the canals if a more decided antiseptic than the oil is needed.—Trueman in *Brief*.

*Care of the Mother's Teeth During Pregnancy.* During that period patients should be instructed, whether they are able to go to the dentist or not, to first keep the teeth absolutely clean by using a good tooth powder or paste, which is natural or slightly alkaline in its action, with a proper tooth brush and antiseptic mouth wash, such as *borine*, *boro-lyptol*, or some similar preparation—and then use freely the milk of magnesia. I have frequently advised patients in that way. For instance, I would lay out a scheme for them as follows: I would say to them, "When you are ready to retire at night, first brush your teeth thoroughly; after your teeth are brushed then take a teaspoonful of milk of magnesia——hold it in your mouth while you are undressing, say five or ten minutes; then when you are ready to get in bed, swallow the milk of magnesia, but do not rinse the mouth afterward—thus the magnesia will lie over the teeth and mucous membrane for several hours and neutralize the acid which oozes out from the gums and lips while sleeping, and dissolves out the lime salts from the enamel. In the morning, upon rising, clean the teeth thoroughly, take a teaspoonful of milk of magnesia, hold it in the mouth and swab it around; keep it there while dressing, then expectorate. In that way they have brushed the teeth at least twice a day and neutralized the secretions with the milk of magnesia at least twice a day and prevented a great deal of trouble. I have had them go through the entire period of pregnancy, and several months afterward, when their teeth would be examined, find scarcely anything to do. You would scarcely know that they had had any trouble whatever. If that method is adopted by the mothers they will reap a great benefit from it.—Dr. S. G. Watkins in March *Items*.

*Care Repaid.* The temporary nature of cement fillings is due in a large measure to the fact that they are regarded as temporary. There are dentists who make their cement fillings last twice as long as those made by other dentists. They regard the operation of filling a cavity with cement as a serious operation, worthy of their highest skill. They are careful in preparing the cavity; they use the best cement and mix it right and insert the material with all the care they give to other operations. Under favorable conditions oxyphosphate fillings—made of the best cement—will last from 3 to 10 years, if inserted as they should be. Under unfavorable conditions, if they are reasonably protected from abrasion, they will last a year or two.

But the profession as a whole has so little faith in any degree of permanency in cement filling that there is but a trifling amount of care given to the work. Failure to get the best results may come from one of three causes: the purchase of an inferior cement, improper chemical combination and mechanical defects.

Regarding the first point, every dentist should realize the vast difference between the qualities of the several cements advertised.

As to the second point, a cement mix is a chemical combination; the liquid is balanced to meet the requirements of the powder. If the mix be too thin or too stiff, or if it is not thorough, the result is not the maximum of strength and endurance. If the liquid bottle is not shaken and certain elements settle to the bottom the operator does not get the result the manufacturer intended and which is possible to get.

Mechanical faults may include insufficient cavity preparation and improper insertion of the filling. When the cement has almost set, in packing it the operator may fold its edges over, leaving flaws in the finished filling that cannot be detected, but which invite the penetration of the dissolving oral fluids.

Fillings of the best oxyphosphate will repay all the skill and care that can be employed.—*Dental Brief.*

*Mental Science and Dental Practice.* The term mental science in the caption is not intended to mean Christian Science, but rather a knowledge of the influences of the mind upon the body, both in health and disease. This influence is not imaginary, but real, all the organs and their functions, all the tissues soft and hard, all the sytsem, including the lymphatic, the nervous and the circulatory, being more or less affected by mental activities. There is, however, but one phase of this deep and most interesting subject to which we desire specially to draw attention, and that deals with the question of lessening the suffering of patients during dental operations.

The marvelous effects of "suggestion" upon hypnotized persons have been recorded by Charcot and others, but few realize that "suggestion" is a potent agency which may be intelligently used with great advantage upon persons in the normal state of wakefulness. Tuke has pointed out that, whereas all persons are subject to sub-conscious influence from suggestion, the result is intensified or accelerated by what he terms "expectant attention." Thus, a cry of "Fire" and the clang and rattle of approaching fire engines causes all people to turn and gaze, whilst with the great majority the impulse to run is almost irresistible. This is a result of suggestion when taken by surprise. But the athlete, standing an his mark, and awaiting the pistol shot which is to start the race, is in a state of "expectant attention," which accounts for the swiftness of the onrush when the "suggestion" to "go" reaches him. Expectant attention is variable, the extremes being pleasurable and painful, and the realization will be tinctured by the intensity of the expectancy. This in part accounts for the great pain experienced during dental operations. There is, of course, a certain degree of pain that perhaps is not preventable, but patients suffer much more than they need; much more than they would if they did not come with dread in their minds; much more than they should, since the dentist, if skilled in mental science, can greatly modify the state of mind in which the patient first sits in his chair. By suggestion he may convince the patient, for example, that "perhaps it will not be so bad after all." By actually avoiding all pain at the first sitting, he can bring his patient back at a second visit without any dread at all, and possibly with the opposite idea that "it is not going to hurt at all." In this stage of expectant attention the operation will really be much less painful.

That the operator's own attitude has much influence upon his patient, who is always in a state of sub-conscious attention toward his least act or word, is well illustrated by the following pretty story:

A tiny tot of a girl was sick, and the doctor was called. He was one of those grave and reverend seignieurs who take life seriously, and save lives in the same manner. The doctor talked long and low to the mother, with many a wrinkle of the brow and shake of the head, till the nervous little patient could endure the suspense no longer:

"Mumsey," she cried, "I goin' to die?"

"Certainly not," replied the startled mother. "Why, what a horrible idea!"

"Then why don't he say it happy?" exclaimed the baby girl.

Why not, indeed? Why not always give our patients the most cheerful, the most hopeful view?

Some years ago the writer formulated a little maxim for the management of children, "Treat children as though they were little men and women"; and more recently the following has been added with equal practical application "and treat men and women as you do little children."—R. Ottolengui in March *Items of Interest.*

*Ammonium Bifluoride.* Having discovered the important fact that hydrofluoric acid would dissolve tartar, without in any way attacking the enamel or cementum, the next step was to modify its action so as to preserve its power of dissolving the tartar and at the same time restrain its caustic and irritating action on the soft tissues of the mouth. The long course of experimentation required to work this out need not be described, nor the accompanying burns received in the process, commented upon. Suffice it to say that the acid salt of ammonium fluoride was at last found to accomplish all that is to be desired. A solution of this salt made as hereafter described, was found to have the power to soften large lumps of tartar so that after one or two applications of the solution they could readily be removed with the scaler, and any microscopic scales were found to be absolutely dissolved, leaving the surface of the treated root smooth and in a receptive condition for the reattachment of the gum. Teeth loosened, where there appeared no irritating tartar in the pockets, also steadily grew firmer and more comfortable under the semi-weekly applications of this solution. When injected into fistulas, the fistulas rapidly healed as the lime salts in the dead bone were rapidly dissolved and the bone cells stimulated to rapidly rebuild the infected area.

The method by which the solution was first made was as follows: Hydrofluoric acid was first neutralized with carbonate of ammonia. This was then filtered. This solution was then evaporated to one-half its bulk, filled up to its original volume with the acid and once more evaporated to one-half its bulk. The resulting liquor was the therapeutic agent spoken of. The evaporation had to be carried on between the temperature of 90 degrees to 105 degrees F., and the method was found applicable to very small quantities only, as in larger bulk the chemical reaction caused an uneven loss of the ammonia and acid, making the final solution either inert or too caustic.

Many manufacturing chemists refused to make it, denying their ability to produce a standard therapeutic solution; but at last the Baker & Adamson Chemical Company, of Easton, were approached and they, with their special apparatus and long experience with the acid in question, found that they were able to produce the standard therapeutic solution at will in any quantity.

Those who have used this bifluoride solution find that their success in the treatment of pyorrhea alveolaris is greatly increased and that mouths which are only moderately affected heal up rapidly and permanently. The bifluoride solution can be allowed to dry on the skin without harmful effect, and yet it will etch glass, forming a rough, frost-like surface. This is a very valuable agent for porcelain inlay workers, making it unnecessary to have hydrofluoric acid about the office, and those who have experienced the appalling septic effect of a hydrofluoric acid burn will consider this in itself no small benefit.

The bifluoride solution when placed in a fistula or in a pyorrhea pocket will only cause a healthy gum stimulation in addition to its power of dissolving calcium salts, but if this solution is allowed to escape into the mouth and dry on the mucous membrane, it makes a burn as bad as that caused by carbolic acid. This latter accident, however, can only occur through gross carelessness, for if the solution is wiped off of the mucous membrane before the expiration of two minutes, no harmful results will occur. The solution should be applied to the fistula or pocket with a rubber syringe with a platinum point. It should always be injected never applied on cotton packing.

In applying the solution the tartar should first be scaled from the root as much as is feasible with scalers, then the gum should be protected on each side of the tooth with napkins or cotton and the pockets filled full. The excess solution should then be carefully wiped dry and new cotton or napkin applied, to avoid the possibility of any of the solution having crept up on the gum or lip by capillary attraction. At the end of two minutes the mouth should be rinsed with water, and when the application has been made to the back teeth, the throat also might be gargled. The applications may be made once a week or twice a week, not oftener. At the end of the first day after the application the gum and tooth will be sore; at the end of the second day it will be more sore; at the end of the third day the gum will be quite well, and on the fourth day a new application can be made.

After a week or two sensitive teeth tend to lose their sensibility to mastication and thermal shocks. The patient will say his mouth feels better. Many pockets heal permanently, and all can be alleviated except when the root has become absolutely necrotic. The bifluoride solution has been in use by its discoverer for two or three years, and he feels that while of itself it will not cure all cases of pyorrhea, properly and carefully used it will largely increase the comfort of patients so afflicted and will multiply the number of absolute cures.—Joseph Head in March *Items*.

